# The specificity of mental pain in borderline personality disorder compared to depressive disorders and healthy controls

**DOI:** 10.1186/s40479-016-0036-2

**Published:** 2016-02-24

**Authors:** Eric A. Fertuck, Esen Karan, Barbara Stanley

**Affiliations:** Department of Psychology, Clinical Psychology Doctoral Program, The City College of the City University of New York, New York, USA; Department of Psychiatry, Columbia University, New York, USA; New York State Psychiatric Institute, New York, NY USA

**Keywords:** Borderline personality disorder, Mental pain, Depression

## Abstract

**Background:**

Individuals with Borderline Personality Disorder (BPD) may experience a qualitatively distinct depression which includes “mental pain.” Mental pain includes chronic, aversive emotions, negative self-concept, and a sense of pervasive helplessness. The present study investigated whether mental pain is elevated in BPD compared to Depressive Disorders (DD) without BPD.

**Methods:**

The Orbach and Mikulincer Mental Pain Scale (OMMP) was administered to BPD (*N* = 57), DD (*N* = 22), and healthy controls (*N* = 31). The OMMP assesses total mental pain, comprised of nine subtypes: irreversibility, loss of control, narcissistic wounds, emotional flooding, freezing, self-estrangement, confusion, social distancing, and emptiness. Co-occurring psychiatric diagnoses, depression severity, and other potentially confounding clinical and demographic variables were also assessed.

**Results:**

The total Mental Pain score did not differentiate BPD from DD. Moreover, most of the subscales of the OMMP were not significantly different in BPD compared to DD. However, the elevation of mental pain subscale “narcissistic wounds,” characterized by feeling rejected and having low self-worth, was a specific predictor of BPD status and the severity of BPD symptoms.

**Conclusion:**

On OMMP total score, mental pain was similarly elevated in BPD and DD. However, the narcissistic wounds sub-type of mental pain was a sensitive and specific diagnostic indicator of BPD and, therefore, may be an important aspect of BPD in need of increased focus in assessment and theoretical models.

## Background

Borderline Personality Disorder (BPD) is a debilitating, and high-risk disorder that is associated with significant emotional suffering and functional impairment [1]. The most frequently co-occurring psychiatric disorders with BPD are Depressive Disorders (DD), most commonly major depressive disorder (MDD) and dysthymia. Between 70 and 90 % of those with BPD go through at least one major depressive episode or exhibit another depressive disorder throughout the course of their lives [2–5]. Moreover, BPD co-occurs for 25 % of those with MDD [6] and dysthymia [7]. However, individuals with BPD may experience a qualitatively distinct form of depression and negative affect. In a review study, Yoshimatu and Palmer [8] concluded that depression in BPD is more chronic form of depression, which shows poor response to the treatment until BPD symptomatology improves. Studies did not show improvement in depression in BPD with treatments such as pharmacotherapy and ECT [9, 10]. This treatment resistant depressive experience is characterized by a high degree of emotional sensitivity that can lead to more frequent or intense and chronic negative affective experiences [11, 12]. These experiences include turbulent aversive affects, negative core beliefs (i.e., that one is “bad” and “worthless”), incoherence of inner experience, hostility/anger, emptiness, a sense of helplessness, loneliness and self-destructiveness as well as fears of abandonment and anxiety [13–16]. The depressive experience in BPD may be distinct, and linked to interpersonal sensitivities and neediness of individuals [16, 17]. Also, BPD patients with MDD are more actively suicidal than the patients with BPD or MDD alone [18, 19]. Although some investigators have proposed that this heightened state of mental pain is a distinguishing and core dimension of BPD [14, 20, 21], there have been few efforts to measure this mental pain in BPD relative to DD without BPD and healthy controls with psychometrically validated instruments.

### Mental pain: theory and measurement

Shneidman [22–24] used the term “psychache” to describe the unbearable mental pain. He argued that this pain is a consequence of unmet psychological needs and the fusion of discrete negative affective state into a generalized mental anguish. Herman [25] and Janoff-Bulman [26] proposed that mental pain is the emergence of a negative sense of self that is caused by trauma and loss. Bolger [27] described this form of psychological suffering as a “brokenness of the self” including the loss of control, loss of self and sense of woundedness while examining the scripts of the people who suffer from traumatic experiences.

Building on these perspectives, Orbach and his colleagues [28, 29] define mental pain as having both affective and self-representational components. According to their model, mental pain is a subjective experience that is distinct from other negative moods and emotions such as depression and anxiety. When an individual’s basic needs are frustrated and there is no anticipated change in the future, basic negative emotional experiences can transform into a more chronic and generalized experience of unbearable mental pain. From this perspective, mental pain is not the same as the more transient negative affects associated with emotional suffering. Compared to transient emotional suffering, mental pain has more trait-like qualities, and is linked to the perception of the self over time. Accordingly, the mental pain concept draws on Bolger’s [27] theory, insofar as it includes a chronic sense of the self as “wounded,” empty, and alienated. To examine the psychometric dimensions of mental pain, Orbach and colleagues have developed a questionnaire to study this integrated construct. The Orbach and Mikulincer Mental Pain Scale (OMMP) measures nine dimensions of mental pain, including irreversibility, loss of control, narcissistic wounds, emotional flooding, freezing, self-estrangement, confusion, social distancing, and emptiness [29]. In an early study using the measure, Orbach and colleagues [28] found a higher level of mental pain on the OMMP in those who had compared to those who had not recently made a suicide attempt. This difference reflected the unique contribution of three mental pain factors: irreversibility, loss of control, and emptiness. Emptiness was the only factor that made a unique contribution to the differentiation between suicidal and non-suicidal participants after controlling for depression, hopelessness, and anxiety. The importance of emptiness and its association with suicidal behavior suggests that the experience of mental pain is a characteristic of BPD [30]. In addition, OMMP scores on all factors except narcissistic wounds and social distancing were significantly negatively correlated with “attraction to life” and positively correlated with “attraction to death” and “repulsion by life” subscales from Multi-Attitude Suicidal Tendencies scale (MAST; [31]).

Another study [29] compared mental pain to depression and anxiety severity among university students and found that all OMMP factors were moderately associated with depressive and anxious cognitions as measured by self-report. This study also reports unique variance of the OMMP factors in predicting these cognitions, suggests that mental pain has distinct facets compared to depression and anxiety. This finding aligns with the clinical and theoretical literature in BPD, which describes that the mental pain experienced by individuals with BPD that are dissociable from the negative emotions associated with depressive disorders [12, 32–34]. While the OMMP has not yet been applied to study BPD, the early uses of the measure suggest that it may be a useful tool for identifying a significant feature of the disorder that has often been overlooked or only indirectly investigated with measures nonspecific to mental pain.

### Clinical models of mental pain in BPD

Historically, BPD researchers and clinicians have emphasized core dimensions such as identity disturbance [35], emotion dysregulation [36], and impulsive-aggression [37]. Mental pain has received less attention, but has also been proposed as a core dimension of BPD [20, 21, 30]. They suggest this multifaceted pain as driving the interpersonal and behavioral symptoms that characterize individuals with BPD. In particular suicidal behavior and non-suicidal self-injury may be a maladaptive strategy for the management and expression of this unbearable state of emotional suffering [20, 30]. Finally, while an expression of negative affect, mental pain may be distinct from the affects and mood symptoms associated with depression and other mood disorders [20].

Though some of the current BPD diagnostic criteria in DSM 5 such as affective instability, chronic feelings of emptiness, intense anger, and recurrent suicidal behavior, suggest an elevation of mental pain, these criteria may not directly capture aspects of mental pain specific to BPD [21]. The negative emotional instability, feelings of emptiness, intense and inappropriate anger, dissociation, and stress related suspiciousness in BPD may be extensions of underlying, chronic mental pain [30].

There are a few studies reporting an association between increased mental pain and BPD. The first study compared individuals with BPD to those with other personality disorders [20] and identified heightened “dysphoria” as a distinguishing feature of the BPD group. Four clusters of states that were characteristic of dysphoria individuals with BPD: extreme feelings, destructiveness, fragmentation or “identitylessness,” and a sense of victimization. In this study, BPD symptoms were associated with feelings of betrayal, urges to self-injure, and feeling out of control. Another study asked clinicians to rate BPD patients they had seen for a minimum of eight sessions using a Q-sort procedure, and found a number of personality traits that were highly characteristic of BPD patients, but that were not captured by the DSM criteria. These included negative affectivity, emotion dysregulation, and self-loathing. The authors suggest that the DSM criteria may underestimate specificity of the dysphoria and mental pain that individuals with BPD experience [34]. Another study of women receiving partial hospitalization treatment [11] found that the increased affect intensity and lower affective control were significant predictors of the number of BPD features after controlling for severity of depression. Finally, in comparing self-report to clinician ratings of depression in BPD compared to MDD without BPD, those with BPD rated their self-report of depression higher than MDD [12]. This contrasted with clinician ratings, which were comparable between groups. Higher scores in self-report depression measures suggest clinicians might not qualitatively differentiate depression experienced by borderline patients from MDD [12]. In aggregate, these studies suggest that mental pain as central to BPD, and that facets of mental pain may not be adequately reflected in the current DSM criteria.

The present study aims to employ the OMMP to evaluate the hypothesis that individuals with BPD will exhibit greater mental pain compared to individuals with DD and HCs. We hypothesized that mental pain would be elevated in BPD relative to DD after controlling for severity of depression and other factors that may influence mental pain such as a history physical or sexual abuse [38, 39] and demographic variables such as sex. We also utilized a HC group to identify which subtypes of mental pain are expressed by individuals with BPD or DD compared to healthy controls.

## Methods

### Participants

All clinical participants were between the ages of 18 and 55 and recruited through a large, metropolitan hospital as part of ongoing clinical studies in mood disorders, suicidal behavior, and BPD. Participants were recruited into one of two different clinical groups. Participants meeting DSM-IV criteria for a diagnosis of BPD and mood disorder without a diagnosis of BPD were recruited. Healthy control (HC) participants between the ages of 18 and 55 were recruited through both the Columbia University Medical Center Department of Psychiatry/New York State Psychiatric Institute (through advertisements) and the John Jay College of the City University of New York’s Psychology Department participant pool. The Institutional Review Boards of both institutions approved the study, all participants were informed about the risks and benefits participation, and all provided written consent.

Exclusion criteria for the BPD and DD groups were schizophrenia and other psychotic disorders, intellectual developmental disorders, history of severe head trauma, or other cognitive impairment that might interfere with the accuracy of assessments or competency to give informed consent. All clinical participants were free of neurological disease as determined by clinical history and examination.

HCs were excluded if they had a current or past Axis I or II psychiatric or substance use disorders after a SCID-I semi-structured interview and SCID-II screener assessment.

### Measures

#### Demographic information

A questionnaire was administered to obtain age, gender, race, education level, and marital status.

#### Clinical assessment

For clinical participants, diagnoses were determined by Structured Clinical Interview for DSM-IV, Patient Edition [40, 41]. Recent reliability studies within our research division yielded the following intraclass correlation coefficients (ICCs) (criterion levels are shown in parentheses): Axis I diagnosis/SCID-I, ICC = 0.80 (0.70); Axis II diagnosis/SCID-II, ICC = 0.70 (0.70); BPD diagnosis, ICC = 0.89 (0.70).

#### Mental pain

The severity of mental pain was measured using the Orbach & Mikulincer Mental Pain Scale (OMMP) [29]. This 44-item scale was first validated in a sample of 255 Israeli university students. Factor analysis of these items revealed 9 subscales that measure the experience of distinct aspects of mental pain, including irreversibility, loss of control, narcissistic wounds/ worthlessness, emotional flooding, freezing, self-estrangement, confusion, social distancing, and emptiness. In the same validation study, the reliability of the OMMP was assessed with test-retest examination with a 3-week interval and a new sample of students (*N* = 53; 30 females and 23 males). The test-retest coefficients for the nine scales ranged between .79 and .94. With regard to convergent validity, scores on all subscales of the OMMP except social distancing were significantly correlated with anxious and depressive cognitions (*r’*s between .26 and .64 for depression and .27 and .50 for anxiety) [29]. OMMP factors were found to be positively associated with coping strategies such as emotion-focused coping, and negatively associated with others, such as problem-focused coping. Orbach and colleagues [29] reported that these associations were moderate, suggesting the scale assesses a subjective experience of mental pain. Thus, it can be significantly differentiated from other measures of distress including the concepts of anxiety and of depression. Another study [42] reported that, among a sample of thirty five individuals who made medically serious suicide attempts and with 67 not serious suicide attempters as well as 71 healthy controls, cronbach alpha coefficients for the factors of OMMP scale ranged between .72 and .89., with the exception of the social distancing factor, which was found to be .42.

#### Depression

Depression severity was rated with the Beck Depression Inventory (BDI) [43] and the Hamilton Rating Scale for Depression HAM-D) [44].

Abuse history was assessed as part of the demographic interview, which asks participants to answer yes or no as to whether they have or have not experienced physical and/or sexual abuse before age 18.

## Data analysis

SPSS Version 18 for Macintosh was utilized for all analyses. For categorical demographic and clinical variables (i.e., sex and race/ethnicity) chi-square analyses were employed to compare groups. Between groups differences on dimensional variables were conducted with ANOVAs comparing the first two clinical groups (BPD and DD) to understand whether there is a significant elevation of mental pain depending on the general psychiatric psychopathology. Then, we compared the three groups BPD, DD, and HC. Subsequently, post-hoc comparisons for significant findings were conducted to address the specificity of the OMMP in predicting BPD status.

## Results

### Demographic and clinical comparisons

There were differences between the BPD, DD, and HC groups on age, sex, and race (see Table 1). Specifically, the mean age for BPD and DD participants was higher than the HCs. Additionally, the percentage of females in the combined clinical group (BPD and DD) was greater than in the HC group. As there was considerable variability in terms of racial and ethnic differences in our groups, we chose to conduct chi-square analysis of racial/ethnic differences by combining racial groups into White and Non-White categories. The BPD group was predominantly White, while the DD and HC groups were predominantly non-White. There were no significant difference between DD and BPD groups in terms of their General Assessment Functioning (GAF) scores. As expected, the clinical groups were both significantly lower from Healthy Controls on their mean GAF scores (see Table 3). Rates of co-occurring disorders for the BPD and DD groups for history of substance abuse or dependence, Post-Traumatic Stress Disorder, Dysthymia, and Panic Disorder were not significantly different between the two clinical groups (see Table 2). Furthermore, 71 % of the BPD group and 65 % of the DD reported that they were on psychiatric medication at the time of assessment.

**Table 1:** Demographic characteristics of BPD, DD, and healthy controls (HC)

	BPD		DD		HC		Analysis	
	(*n* = 57)		(*n* = 22)		(*n* = 31)		*p* ^a^	Contrast^b^
	M	SD	M	SD	M	SD		
Age (years)	32.1	9.1	34.6	10.0	25.6	11.2	.003	BPD, MDD > HC
Education (years)	14.9	2.1	13.8	2.8	14.7	1.2	.106	
Race/ethnicity	*N*	%	*N*	%	*N*	%		
Caucasian	34	59.6	4	18.2	10	32.3		
Black	5	8.8	7	31.8	10	32.3		
Asian	2	3.5	0	0.0	1	3.2		
Hispanic	7	12.3	6	27.3	10	32.3		
More than one race	1	1.8	2	9.1	0	0.0		
Unreported or unknown	8	14.0	3	13.6	0	0.0		
White vs. non- white	34	59.6	4	18.2	10	32.3	.001	
Sex								
Female	50	87.7	15	68.2	17	54.8	.002	

^a^ANOVA for continuous variables; chi-square analyses for categorical variables

^b^Post-hoc comparisons

**Table 2:** Axis I and axis II diagnosis of BPD and DD

	BPD		DD	
	*N*	%	*N*	%
Axis I diagnoses				
Panic disorder	8	14.0	4	18.2
Agoraphobia without panic disorder	3	5.3	1	4.5
Simple phobia	7	12.3	3	13.6
Generalized anxiety	6	10.5	1	4.5
Social phobia	7	12.3	4	18.2
Obsessive compulsive disorder	12	21.1	0	0
Post traumatic stress disorder	17	29.8	4	18.2
History of substance abuse/dependence	29	50.9	11	50.0
Current substance abuse/dependence	8	14	1	4.5
Major depression	48	84.2	18	59.1
Current major depression episode	28	49.1	5	22.7
Bipolar I	3	5.3	1	4.5
Current mood episode	2	3.5	1	4.5
Bipolar II	4	7.1	3	13.6
Current mood episode	2	3.5	3	13.6
Dysthymia	6	10.5	3	13.6
Depressive disorder NOS	1	1.8	0	0
Eating disorder	11	19.3	0	0
Binge eating disorder	7	12.3	0	0
Attention deficit hyperactivity disorder	1	1.8	0	0
Axis II diagnoses				
Paranoid	4	7.1	0	0
Schizotypal	4	7.1	0	0
Obsessive-compulsive	6	10.5	3	13.6
Histrionic	0	0	0	0
Dependent	2	3.5	0	0
Antisocial	2	3.5	3	13.6
Narcissistic	4	7.1	1	4.5
Avoidant	8	14.0	1	4.5
Passive-aggressive	5	8.8	1	4.5

### Comparisons between BPD, DD, and HCs on mental pain, depression, and hopelessness

As a first step, we tested whether the OMMP was elevated in the combined clinical group relative to HCs. ANOVAs demonstrated significantly higher scores in the combined clinical group (BPD and DD) compared to controls on all factors of mental pain except “social distancing” (Table 3). Next, we compared the three groups. ANOVAs indicated that the BPD group scored significantly higher than both DD and HC on depression severity (HAM-D) and hopelessness (BDHI). Also, the groups differ in regard to age, sex, and race; we used these variables as covariates. MANCOVA allows for inclusion of covariates with multiple continuous dependent variable analysis. We conducted the analysis for each continually measured item of the OMMP. Thus, a subsequent MANCOVA controlling for sex, age, race and depression severity and hopelessness was conducted and demonstrated that only the “narcissistic wounds” mental pain factor was more elevated in BPD participants as compared to both the DD and control groups (*p* = .002).

**Table 3:** Mental pain subscales and other clinical variables of BPD, DD, and HC

Rating scales scores	BPD		DD		HC		Analysis	
	M	SD	M	SD	M	SD	*p* ^a^	Contrast^b^
GAF score	58.59	9.15	62.25	6.91	84.11	4.68	.00	BPD, DD > HC
Mental pain total score	121.8	31.1	105.9	37.2	67.5	16.0	.00	BPD, DD > HC
Mental pain subtypes:								
Irreversibility	23.56	8.26	23.14	8.81	12.77	4.50	.00	BPD, DD > HC
Loss of control	27.95	9.40	22.14	9.54	13.52	3.92	.00	BPD, DD > HC
Narcissist wounds	13.26	4.35	9.50	5.09	6.61	2.00	.00	BPD > DD > HC
Emotional flooding	15.23	3.62	13.36	4.98	7.03	3.01	.00	BPD, DD > HC
Freezing	6.96	3.12	5.59	3.17	3.55	1.00	.00	BPD, DD > HC
Self-estrangement	7.77	2.88	6.68	3.37	4.23	1.63	.00	BPD, DD > HC
Confusion	9.21	3.39	8.55	3.71	4.87	1.88	.00	BPD, DD > HC
Social distancing	10.89	2.85	11.05	2.75	11.35	1.40	.71	
Emptiness	6.91	3.09	5.91	3.18	3.55	1.18	.00	BPD, MDD > HC
Beck depression inventory	18.9	9.7	13.9	10.8	2.7	3.0	.00	BPD, MDD > HC
Hamilton depression scale	14.1	8.3	10.2	7.2	0.0	0.0	.00	BPD, MDD > HC
Scale of suicidal ideation (current = past week)	3.8	6.0	1.0	2.4			.09	
	*N*	%	*N*	%				
Physical or sexual abuse before age 18	28	50.9	10	49.1			.58	
Suicide attempter	31	53.39	17	77.27			.08	

*N* = 109 for the Beck Depression Inventory due to missing data from one BPD participants

*N* = 88 for the Hamilton Depression Scale due to missing data from 1 MDD and 21 HC participants

*N* = 79 for Scale for Suicidal Ideation (*N* = 56 for BPD and *N* = 22 for MDD)

^a^ANOVA for continuous variables; chi-square analyses for categorical variables

^b^Post-hoc comparisons

### The specificity of the “narcissistic wounds” mental pain factor as a predictor of BPD symptom severity

To further clarify the specificity of the narcissistic wounds subscale to severity of BPD symptoms, we conducted a series of stepwise, linear multiple regression analyses. First we evaluated the predictive impact of demographic variables on the number of SCID-II BPD symptoms, irrespective of diagnostic group. Abuse history, age, education, and marital status did not predict number of BPD symptoms. However, White vs. Non-White status (b = −.23, *t*(78) = 2.20, *p* < .05) and sex (b = .22, *t*(78) = 2.03, *p* < .05) independently predicted the number of BPD symptoms among these demographic variables. Next, we conducted a multiple regression analysis in blocks, with the first block controlling for White vs. Non-White status and sex. In the second block we included the Hamilton Depression Score and the total Beck Hopelessness score to control for depression severity and hopelessness. In the final block, we added the narcissistic wounds mental pain sub-score. In this regression, only the White vs. non-White (b = −.22, *t*(78) = −2.00, *p* < .05) and higher narcissistic wounds scores predicted a greater number of BPD symptoms (b = .28, *t*(78) = 2.56, *p* < .05), indicating that Beck Hopelessness and Hamilton Depression scores are not uniquely predictive of BPD symptoms. The final model then, we included only White vs. non-White and narcissistic wounds as independent variables, with White vs. non-White force entered first in the first block. In this final model, higher narcissistic wounds predicted unique variance in predicting a greater number of BPD symptoms above White vs. non-White ethnicity status (semi-partial correlation [pr] = .35, b = .35, *t*(78) = 3.38, *p* < .001).

## Discussion

The present study did not find an elevation of mental pain total on the OMMPS in BPD relative to DD without BPD. Concurrently, eight of the nine mental pain subscales were not significantly different between BPD and DD, suggesting that most dimensions of mental pain measured by the OMMPS are expressed similarly in BPD and DD. However, the “narcissistic wounds” mental pain subtype, which measures the experience of rejection and loss in conjunction with a devaluation of the self, was the only subscale that was associated with BPD status and severity of BPD symptoms, independent of depression status or severity. Moreover, there was no difference on GAF scores between BPD and DD, overall psychopathology is unlikely to drive OMMP differences between BPD and Dd. The results of the present study, then, provide partial support for models of BPD that posit mental pain as a dimension that is unique to BPD.

While mental pain is a promising construct in psychopathology and suicide research, the role of most of the dimensions of mental pain on the OMMP was not in BPD relative to depressive disorders. This may indicate that the mental pain scale is not sensitive to the qualitatively different depression that has been described among individuals with BPD. Alternatively, the results might suggest the common pathology and high co-occurrence between BPD and DD [45]. For instance, mental pain could, rather, be a trait underlying suicidality and non-suicidal self-injury in BPD and DD. Of relevance to mental pain, longitudinal studies have highlighted that the affective dimensions of BPD are resistant to change relative to other symptoms such as impulsivity and suicidality [3]. Therefore, mental pain may be an important and challenging symptom dimension that cuts across diagnostic categories, and may also be germane to recurrent depressive disorders without BPD [46]. In this way, mental pain may be a trait like negative affect that neighbors other negative emotional traits that are elevated in BPD and DD.

The one subscale that differentiated BPD from DD was narcissistic wounds. This subscale may include items that tap rejection sensitivity [38, 39, 47] and shame proneness [48–51]. For example, the five Items from “narcissistic wounds” subscale of Mental Pain questionnaire include items that focus on the feeling that one is of no value to oneself or others. These items include, “Nobody is interested in me,” “I am rejected by everyone,” “I feel abandoned and lonely,” “Others hate me,” and “I am worthless.” With the exception of the abandonment criterion of the DSM-5, the low self-worth and pervasive sense of depreciation from others is not clearly represented in the current DSM criterion set. This subscale speaks to a highly devalued self-concept co-occurs with a belief that one is depreciated by invisible to significant others. Furthermore, Zanarini and her colleagues [20] found that feeling “worthless” was more common in BPD patients although it was not specific to BPD compared to patients with other personality disorder. At the same time, the belief that “people hate me” and the feeling that one is “abandoned” were specific to BPD compared to other personality disorder group [20]. The DSM-5, Section III has included “Disturbances in the self and interpersonal functioning” as an essential feature of personality disorders. “Malignant Self-Regard” [52] defined by a highly self-critical attitude towards the self, paired with unrealistic expectations for the self (i.e., perfectionism), may be associated with to the narcissistic wounds aspect of mental pain we have identified. While this requires further investigation, the direction of the DSM towards dimensional models of the self in relation to others is consistent with the highly specific correlation between narcissistic wounds and BPD features we report.

### Limitations

We assessed mental pain with a self-report questionnaire, which is subject to a range of psychometric issues, including social desirability influences [53], and variability in accuracy of self-assessment due to emotional state arousal and severity of psychopathology [12]. Additionally, the high rates of co-occurring depressive disorders and high levels of depressive severity in the clinical sample may have obscured aspects of mental pain that are unique to BPD. Future studies could include those with BPD and no current depressive disorder compared to those with remitted depression to further investigate this possibility. Moreover, the studies of mental pain need to move beyond self-report instruments and include laboratory [54] and psychobiological methods [55]. Ecological momentary assessment [56] can capture the immediate precipitants and contextual factors associated with mental pain. Our sample of healthy participants was not matched on sociodemographic variables, which may have contribute to a source of error variance even though the differences between samples on such demographics were statistically controlled. Lastly, due to our limited sample size, we may not have had the power to detect the small to medium effect size differences between BPD and DD. A future replication study in a larger and more representative sample will be needed to further understand the construct of mental pain in various types of psychopathology.

## Conclusions

Mental pain, as assessed by the total score of the OMMPS, is was not higher in BPD compared to DD without BPD. However, one aspect of mental pain that BPD exhibits that was unique was a perception of the self as worthlessness depreciated and an experience of rejection from others (i.e., the “narcissistic wounds” subscale). The degree to which mental pain is specific to forms of psychopathology, or, behaviors such as suicide and NSSI is an important area of future investigation [28, 57].

## Abbreviations

BDI: Beck Depression Inventory
BHS: Beck Hopelessness Scale
BPD: Borderline Personality Disorder
DD: Depressive Disorders
DSM: Diagnostic Statistical Manual
ECT: Electroconvulsive Therapy
HAM-D: Hamilton rating scale for Depression
HC: Healthy Controls
MAST: Multi-attitude Suicidal Tendencies Scale
MDD: Major Depressive Disorder
OMMP: The Orbach and Mikulincer Mental Pain Scale
SCID-I: the structured clinical interview for DSM-IV Axis I Disorders
SCID-II: the structured clinical interview for DSM-IV Axis II Disorders
SPSS: statistical program software package
